# High prevalence of *mcr-1*-encoded colistin resistance in commensal *Escherichia coli* from broiler chicken in Bangladesh

**DOI:** 10.1038/s41598-020-75608-2

**Published:** 2020-10-29

**Authors:** Shahana Ahmed, Tridip Das, Md Zohorul Islam, Ana Herrero-Fresno, Paritosh Kumar Biswas, John Elmerdahl Olsen

**Affiliations:** 1 Department of Veterinary and Animal Sciences, Faculty of Health and Medical Sciences, University of Copenhagen, Frederiksberg, Denmark; 2 Department of Microbiology and Veterinary Public Health, Chattogram Veterinary and Animal Sciences University, Chattogram, Bangladesh; 3 Section on Pathophysiology and Molecular Pharmacology, Joslin Diabetes Center, Boston, MA USA; 4 Department of Microbiology and Immunobiology, Harvard Medical School, Boston, MA USA

**Keywords:** Antimicrobials, Applied microbiology, Bacteria, Bacteriology, Clinical microbiology, Microbial communities, Infectious-disease diagnostics, Policy and public health in microbiology, Microbial ecology, Computational science, Scientific data, Statistics, Ecology, Microbiology, Molecular biology, Diseases, Risk factors, Evolutionary biology, Functional genomics, Genome, Genomics, Microbial genetics, Mutation, Sequencing

## Abstract

Colistin is a last-resort antimicrobial used for the treatment of human infections caused by multidrug-resistant Gram-negative bacteria. However, colistin is still widely used in intensive poultry production in Bangladesh. We aimed to investigate the dynamics and genetic diversity of colistin-resistant commensal *Escherichia coli* from broiler chickens. A total of 1200 *E. coli* strains were characterized from 20 broiler farms at three-time points along the production period. All strains were screened for *mcr-1* to *mcr-5* genes by a multiplex PCR, and their genetic diversity was measured by repetitive extragenic palindromic (REP)-PCR fingerprinting. Genomic diversity and characterization were performed by whole genome sequencing (WGS). Twenty-five percent of the commensal *E. coli* strains harbored *mcr-1* genes. Frequency of *mcr-1* gene detection correlated positively (odds ratio 1.71; 95% CI 0.96–3.06; *p* = 0.068) with the use of colistin in poultry flocks. REP-PCR profiles and WGS analysis showed diverse *E. coli* population carrying multiple antimicrobial resistance genes. Phylogenetic comparison of *mcr-1*-bearing strains recovered from this study with a global strain collection revealed wide phylogenetic relationship. This study identified a high prevalence of *mcr-1* gene among genetically diverse *E. coli* populations from broiler chickens in Bangladesh suggesting a massive horizontal spread of *mcr-1* rather than by clonal expansion.

## Introduction

The gut of warm-blooded animals is the primary habitat to *Escherichia coli,* and the type of relationship between *E. coli* and its host is mainly that of commensalism. The diversity of the commensal *E. coli* populations is influenced by many factors such as host species, environment, age of the host, type of food and antimicrobial treatment^1–3^. There have been many studies on pathogenic *E. coli*, while little is known about the commensal population. Understanding the genetic background and population structure of commensal *E. coli* is necessary to explore their potentials as a reservoir of antimicrobial resistance (AMR) determinants and virulence factors^4^. Use of antimicrobials as therapeutic agents or growth promoters affects the commensal population as it kills or reduces the growth of susceptible strains, giving advantage to strains that have become resistant, e.g. by the acquisition of resistance genes by horizontal gene transfer^5,6^.

AMR in *Enterobacteriaceae* has become a global health concern. It is well documented that the use of antimicrobial in food animal production is a possible source of AMR in humans through horizontal transfer of either antimicrobial resistance genes (ARGs) to human pathogens or through direct transfer of AMR bacteria^7,8^.

Colistin is one of the most commonly used antimicrobials in livestock, especially in developing countries like Bangladesh^9^, which typically lack strong regulation for antimicrobial use (AMU). Resistance to colistin is worrisome because the drug is considered a last resort for the treatment of serious infections caused by carbapenem-resistant organisms belonging to the family *Enterobacteriaceae*^10^. Before 2015, resistance to colistin was only known to be caused by chromosomal mutations. Later, plasmid-mediated colistin-resistance has been reported from many countries, and to date, nine *mcr* genes (*mcr-1* to *mcr-9*) have been discovered along with some variants^11–19^.

In Bangladesh, small-scale broiler farms are the major source of poultry meat. Colistin sulphate is used massively for the treatment and prevention of diseases in this production system^9^. Owing to the absence of strict regulation for AMU in Bangladesh, farmers can acquire colistin without prescription from a registered veterinarian^9^. To date, there is no systematic investigation on the colistin resistance commensal *E. coli* population in animals in this production system where imprudent use of antimicrobial is very frequent. Moreover, the overall genetic diversity of the commensal *E. coli* population in broiler chicken over the entire production stage has not been investigated before.

In this study, the genetic diversity of commensal *E. coli* from broiler chicken in Bangladesh was thoroughly investigated throughout the production period, and the prevalence of plasmid-encoded colistin resistance genes (*mcr*) was determined. The objectives of the current study were to investigate the genetic diversity of commensal *E. coli* in poultry, to explore the distribution and genetic background of colistin-resistant strains among the commensal *E. coli* population, and to characterize the phylogenetic relationship of the colistin-resistant commensal *E. coli* strains in the global population structure of *E. coli*.

## Results

### Farms, sampling and *E. coli* isolates

A total of 20 commercial broiler chicken farms at Chattogram division in Bangladesh were investigated in this study. Farms were located across two administrative districts of the division with a minimum and maximum distance between farms of 0.5 km and 80 km, respectively. All the birds were raised in small-scale intensive systems of rearing. The farm size varies from 800 to 4000 birds per farm (median size 1060 birds/farm). Most farms used antimicrobials for treatment and prophylaxis purpose. No farms used antimicrobials for the purpose of growth promotor. A detail list of different classes of antimicrobials used during the production is given in Supplementary Table 1.

**Table 1:** Univariable and multivariable analysis for the risk factors associated with the prevalence of *mcr-1* gene in commensal *E. coli* population.

Variable	Co-variables	*mcr-1*/total *E. coli (%)*	Univariable		Multivariable	
			Odds ratio (95% CI)	*P* value	Odds ratio (95% CI)	*P* value
Antimicrobial use^a^	Yes	244/660 (36.8)	1.77 (0.95–3.29)	0.070	1.19 (0.57–2.49)	0.640
	No	61/540 (11.3)	–	–	–	–
Colistin use^a^	Yes	171/340 (50.3)	1.85 (1.15–2.98)	0.011	1.71 (0.96–3.06)	0.068
	No	134/860 (15.6)	–	–	–	–
Broiler strains	Lohmann	26/60 (43.3)	3.63 (0.46–28.91)	0.223	–	–
	Ross-308	36/120 (30.0)	1.72 (0.38–7.83)	0.483	–	–
	Cobb500	243/1020 (23.8)	–	–	–	–
Water source	Tube well	214/840 (28.7)	1.02 (0.24–4.26)	0.983	–	–
	WASA	2/60 (3.3)	0.08 (0.01–1.15)	0.064	–	–
	Pond	43/120 (35.8)	2.07 (0.32–13.47)	0.446	–	–
	Mixed source	13/60 (21.7)	0.88 (0.09–8.78)	0.913	–	–
	Deep water	33/120 (27.5)		–	–	–
Footbath	Spray	256/1080 (23.7)	0.32 (0.07–1.41)	0.131	–	–
	No	49/120 (40.8)	–	–	–	–
Floor	Muddy	160/660 (24.2)	0.90 (0.34–2.36)	0.825	–	–
	Concrete	145/540 (26.9)	–	–	–	–
Ventilation	Moderate	153/480 (31.9)	2.03 (0.81–5.11)	0.133	–	–
	Good	152/720 (21.1)		–	–	–

*CI* confidence interval, *WASA* Water Supply and Sewerage Authority.

^a^Use of antimicrobials within 14 days prior to sampling.

One pooled faecal sample were collected in each of the three sampling times at day1, day15, and day28 of the production which comprises 60 pooled samples from 20 farms.

**Figure 1:**
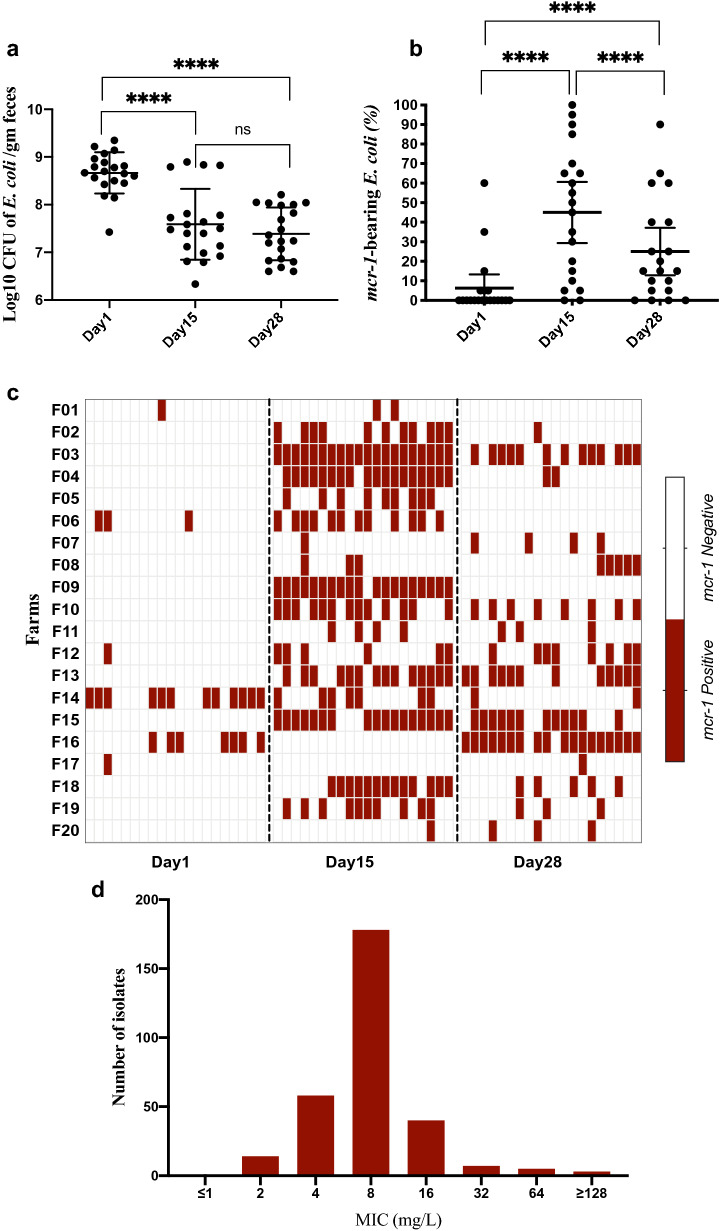
Quantitative detection, phenotypic and genotypic confirmation of colistin-resistant strains among the commensal *E. coli* population. (**a**) Log_10_ transformed colony forming units (CFU) of commensal *E. coli* per gm faeces over the three-sampling time. The Y axis was scaled down to the data point. Each black dot represents one pooled faecal sample and the bars represent mean and standard deviation. The asterisk denotes *p*-value of unpaired *t*-test between sampling times, ****p < 0.0001, ns = non-significant. (**b**) Frequency of *mcr-1*-bearing strains among the commensal *E. coli* population. The frequency was calculated by dividing the number of *mcr-1*-positive *E. coli* with the total number of *E. coli* strains*.* Each black dot represents one pooled faecal sample and the bars represent mean and standard deviation. (**c**) Heatmap shows the distribution of *mcr-1*-bearing *E. coli* across the farms and sampling time. The rows denote farms and the columns denote individual *E. coli* isolate over three sampling times. (**d**) Minimum Inhibitory Concentration (MIC) distributions of *mcr-1*-bearing *E. coli* (N = 305) against colistin sulphate as determined by a broth microdilution technique.

Quantification of *E. coli* from faecal specimens shown that the average count of *E. coli* was significantly (*p* < *0.0001*) higher at day1 (8.7 ± 0.43 log_10_ CFU/gm faeces) compared with day15 (7.6 ± 0.74 log_10_ CFU/gm faeces) and day28 (7.4 ± 0.55 log_10_ CFU/gm faeces) (Fig. 1a). A total of 20 confirmed *E. coli* isolates from each faecal sample, comprising an overall total collection of 1200 isolates, were characterized in this study.

### A high prevalence of *mcr-1* gene was detected in the commensal *E. coli* population

We identified 305 (25%, 95% CI, 23–28%) *mcr-1*-positive *E. coli* out of the 1200 isolates. No strains were positive for the other genes (*mcr-2* – *mcr-5*) investigated. The prevalence of the *mcr-1* gene in the *E. coli* population was significantly higher (*p* < 0.0001) at day15 (45%) compared with day28 (25%) and day1 (6%) in the production (Fig. 1b). The distribution of *mcr-1*-bearing *E. coli* isolates across the farms at three sampling times was highly diverse (Fig. 1c). Colistin-resistant *E. coli* could be detected in all farms at least one sampling time. Seven variables were tested in the univariable analysis (Table 1). Of them, two parameters: “antimicrobial use” and “colistin use” were identified as eligible to be included for the multivariable analysis. In the final model “colistin use” was found to be a potential risk factor (OR 1.71; 95% CI 0.96–3.06; *p* = 0.068) associated with the occurrence of *mcr-1* gene in the commensal *E. coli* population (Table 1).

### MIC shows an extreme level of resistance against colistin in some strains

The MIC values of *mcr-1-*positive isolates varied from 2 to 128 mg/L. Notably, more than 50% of the isolates had a MIC of ≥ 8 mg/L (Fig. 1d). On the other hand, none of the *mcr-1*-negative isolates had MIC above the breakpoint.

### REP-PCR based genetic diversity

In total, 367 unique REP-types were identified among the 1200 *E. coli* isolates (Supplementary Table 2). Of them, 89 (24%) types were present both in *mcr*-positive and *mcr*-negative *E. coli*. In total, 50 (14%) and 228 (62%) unique REP-types were found in *mcr*-positive and *mcr*-negative *E. coli*, respectively. High overall diversity was observed as shown by the Shannon diversity index (*H'*) of 5.3. The highest diversity was found in the sample of day28 with an *H'* of 4.9 followed by day15 (*H'*, 4.2) and day1 (*H'*, 4.1). However, the genetic diversity among the *mcr*-negative *E. coli* strains was slightly higher (*H'*, 5.3) compared with the *mcr*-positive *E. coli* (*H'*, 4.6).

### Genome assembly and annotation

In total, 32 *E. coli* draft genomes were produced and assembled using a hybrid assembler Unicycler. The median length of the assembly was 5.06 Mbp with an average GC% of 50.62 ± 0.2. The average N50 of the assembled contigs was 56 Kbp. Annotation of the 32 draft *E. coli* genomes predicted a median number of 4819 coding sequences (CDs) (ranging from 4102 to 5227). The median number of contigs per assembly was 300 with a minimum of 151 and a maximum of 664 (Supplementary Table 3).

### Core and pangenome comparison

**Figure 2:**
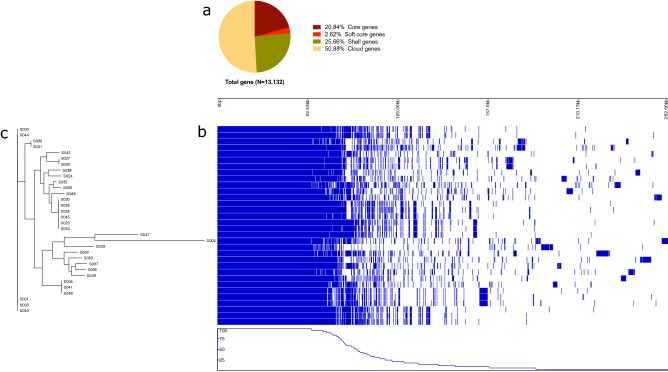
Pangenome comparison of *mcr-1*-positive commensal *E. coli*. (**a**) Distribution of total genes: core genes when found in ≥ 99%, soft-core genes when found between 95 and 99%, shell genes when between 15 and 95% and cloud genes when found in < 15% of the isolates. (**b**) Gene presence (blue) absence (white) matrix across the genome of the isolates. Top scale shows the comparative genome size. Each row represents the gene content of the respective isolate. Each column represents the possession of homologous gene clusters. A complete list of annotated genes is provided in the Supplementary File 2. The bottom graph shows percent similarity of gene presence-absence between isolates. (**c**) Maximum likelihood phylogenetic tree inferred from 2737 concatenated core gene alignment of 32 commensal *E. coli* genome. The tree was constructed by RAxML with 1000 bootstrap using GRT + gamma model. The data was visualized using Phandango.

The degree of genomic flexibility of the 32 *mcr-1*-positive commensal *E. coli* strains was assessed by comparing the core and pangenome structure of the isolates. The overall pangenome consisted of 13,132 genes which is four times larger than the core genome of the same strains (2737 genes (20.84%) (Fig. 2a). The accessory gene pool was highly variable among the isolates (N = 10,395). Of the genes identified by Roary, 50.88% (n = 6681), 25.66% (n = 3370), and 2.62% (n = 344) were found in less than 15% of the isolates (referred to as cloud genes), between 15 and 95% of the isolates (referred as shell genes), and between 95 and 99% of the isolates (referred as soft core genes), respectively (Fig. 2a). We compared the genome of *mcr-1*-positive *E. coli* isolates based on the presence or absence a gene which is depicted in Fig. 2b). Phylogenetic comparison based on the concatenated core gene alignment also showed large genomic diversity among the *mcr-1*-positive commensal *E. coli* isolates (Fig. 2c).

### Virulence genes harboured by commensal *E. coli*

In Total, 13 virulence genes were detected in the genomes of the 32 commensal *E. coli* (Supplementary Table 4). The most frequent virulence determinants were *astA* (EAST-1 heat-stable toxin) and *iss* (Increased Serum Survival) which were found in 50% and 44% of the isolates, respectively. A major proportion of the isolates (53%) harboured multiple virulence genes. Notably, seven of the 32 isolates harboured at least 4–6 virulence determinants, but none of the strains carried combinations of virulence genes known to be characteristic for pathogenic subtypes.

### In silico MLSTs, serotypes, and plasmid genotyping

We identified 16 different sequence types (STs) among the 32 *E. coli* isolates. The most frequent ST was ST43 (n = 6) followed by ST4965 (n = 5). We could not infer the ST types of three isolates (listed as unknown types in Supplementary Table 4). In silico serotyping identified 11 complete serotypes. The most frequent serotype was O13:H30 (n = 6) followed by O6:H10 (n = 4). We could not identify the O antigen specific serotype of 11 of the isolates. However, based on the H type, three isolates were O-:H10 and two were O-:H21 and seven were singletons. We identified 31 plasmid replicons in the 32 isolates. All the isolates harboured multiple plasmid replicons ranging from four to 11. The most abundant replicon type was ColRNAI which was found in 24 isolates followed by IncFIB (AP001918) in 23 isolates. Other dominant plasmid replicon types were IncHI2 (n = 19), IncHI2A (n = 19), IncN (n = 16), IncX1 (n = 15), and IncI2 (n = 13).

### High prevalence of antimicrobial resistance determinants

**Figure 3:**
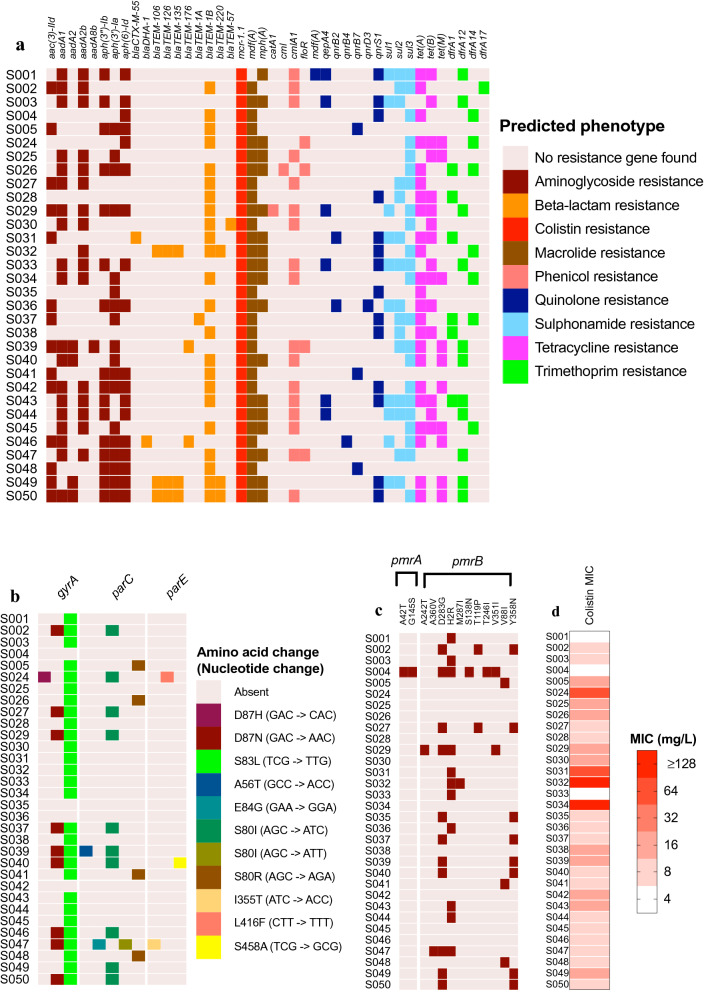
Antimicrobial resistance determinants identified in the genomes of *mcr-1-*positive commensal *E. coli*. (**a**) Heatmap shows the presence or absence of antimicrobial resistance determinants of commensal *E. coli*. The rows represent isolates and the columns correspond predicted antimicrobial resistance genes (ARGs). ARGs are predicted from whole genome sequence data using Resfinder 3.2 tool on CGE webserver. (**b**) Heatmap represents chromosomal known point mutation in the commensal *E. coli* genome. Colours indicate amino acid substitution at the corresponding mutation site. Rows and columns represent isolates and mutated genes, respectively. (**c**) Point mutations in *pmrA* and *pmrB* genes of commensal *E. coli*. Columns and rows represent amino acid substitution position and individual isolates, respectively. Dark and light blocks show the presence and absence of mutation, respectively. (**d**) Distribution of minimum inhibitory concentration (MIC) against colistin sulphate among *E. coli* strains. Rows and columns represent strains and MIC, respectively.

We identified a total of 42 different antimicrobial resistance determinants in the 32 strains (Fig. 3a). The strain selection was based on the presence of *mcr-1* and in accordance, this gene was detected in all 32 strains. The most abundant antimicrobial resistance gene was *mdf*(*A*) (31/32) followed by *tet*(A) (23/32) and *bla*_TEM-1B_ (23/32) which confer resistance to a broad spectrum of antimicrobials, tetracycline, and beta-lactam antibiotics, respectively. All 32 strains carried multiple acquired antimicrobial resistance genes with a minimum of five to a maximum of 20 genes (Fig. 3a). According to the database used, more than 50% of the strains (n = 18) harboured at least 13 resistance genes.

Twenty-seven of the isolates exhibited at least one known point mutation and 15 isolates exhibited at least two point mutations in any of the three-resistance determinants (*gyrA*,* parC*, and *parE*) which may confer resistance to nalidixic acid and ciprofloxacin (Fig. 3b). The insertion sequence IS*Apl1* was found in 30 of the 32 *mcr-1*-bearing *E. coli* strains (Supplementary Table 4). The other two had no IS*Apl1* insertion sequence in their genomes. A total of eight, fifteen, and six of the isolates contained one, two, and three IS*Apl1* insertion sequences, respectively.

### Presence of chromosomal point mutations in genes previously associated with colistin resistance

Since MIC varied considerably between *mcr-1-*positive isolates, we carried out a search for point mutations known to be associated with elevated MIC towards colistin in *E. coli*. No known colistin resistance associated point mutations in *pmrA* and *pmrB* genes was detected among the 32 *mcr-1*-carrying isolates. However, 22 out of 32 *mcr-1*-positive strains showed point mutations in *pmrA* and *pmrB* genes (Fig. 3c). A side-by-side comparison with point mutations and the MIC value of these 32 *mcr-1*-positive strains are shown by a heatmap in Fig. 3d.

### Phylogenetic position of *mcr-1*-positive *E. coli* at the global population structure

**Figure 4:**
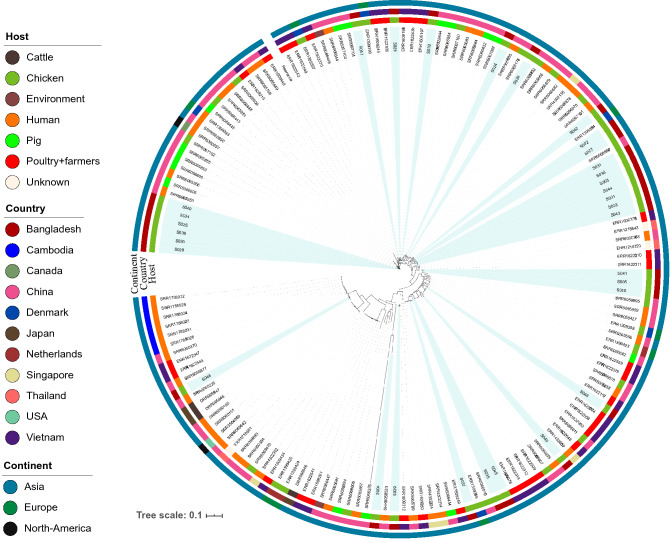
Maximum-likelihood phylogeny based on concatenated core genome SNPs of 158 *mcr-1*-bearing *E. coli* strains. The phylogeny was inferred from a total of 93,130 single-nucleotide polymorphisms (SNPs), of which 52,636 were parsimony-informative and 40,494 were singletons. Isolates from this study are highlighted with colour shade. *E. coli* K12-MG1655 was used as a reference strain.

The WGS of *mcr-1-*positive strains identified in the current study were compared to a global collection originated from Cambodia (6 human isolates), Canada (one human isolate), China (two environment, 16 chicken, 33 human, and 15 pig isolates), Denmark (one human and five chicken isolates), Japan (three cattle isolates), the Netherlands (three chicken isolates), Singapore (five human isolates), Thailand (three isolates from unknown source), the USA (one human isolate), and Vietnam (32 isolates from poultry farms and farmers) (Supplementary File 1). A total of 93,130 core SNPs was detected among the 158 isolates. The pairwise comparison of SNP between isolates varied from 1 to 46,621 SNPs. A total of six and five *E. coli* isolates from this study, respectively, formed two separate clades. However, most of the strains of this study were spread across the phylogenetic tree (Fig. 4).

## Discussion

Colistin sulphate is widely used for treatment and for the prevention of diseases in poultry in Bangladesh^9^. Poultry farmers in Bangladesh usually administer colistin or other antimicrobials through the water, and often in the absence of disease symptoms. Once they start using any antimicrobials treatment, they continuously keep it in water for a certain period of time ranging from three to seven days. Little is known on how this imprudent use of antimicrobial over the decades have affected the genetic diversity of commensal gut bacteria in the poultry.

Our study detected a diverse population of commensal *E. coli* in broiler chicken according to genetic typing. The genetic diversity of *E. coli* increased with the age of the birds. Previous studies^20–22^ have also shown the association of age of host (e.g., pigs) with the diversity of commensal *E. coli*. The increased diversity in the older age group of chicken might be due to the establishment and persistence of adapted strains in the gut over time.

Apart from the wide genetic diversity, we detected a high prevalence of the *mcr-1* gene (25%) in the commensal *E. coli* population. Although the study design and the host species were different, a similar alarming high frequency of *mcr-1* was detected in *E. coli* from human and livestock sources in Thailand^23^ and one study from China^24^. Overall, the high frequency of *mcr-1* gene detected in this study is still the exception, and much higher than previously reported studies from Germany (3.8%)^25^, China (1% or less) and other countries^26–28^. Also, this study shows high MIC against colistin (≥ 8 mg/L) in more than 50% of the *mcr-1* carrying *E. coli* which is very similar with another study in Bangladesh^29^, and the overall MIC is higher than other studies in some European countries^25,30,31^. It suggests that the commensal *E. coli* population in livestock in countries with a high and unregulated use of colistin constitutes a hotspot for selection for carriage of transferrable resistance. This is worrisome and calls for immediate action to reduce the preventive use of antimicrobials in general and colistin in particular. The study showed that the occurrence of the *mcr-1* gene increased significantly as the birds grew older, most likely simply reflecting the selection pressure. In accordance with this, colistin use is positively associated with the isolation frequency of the *mcr-1* gene in this study. This association should be cautiously interpreted as the selection and spread of AMR gene is a complex biological process. Although the selection of *mcr-1* gene is primarily influenced under the pressure of colistin, other antimicrobials can also co-select *mcr-1* gene^32^. Additionally, many factors, such as habitat, co-selection by metals, and biocides, can influence the selection of antimicrobial resistance genes^33^. There is no veterinary monitoring of antimicrobial usage for the studied farms, therefore, we mainly relied on the data provided by the farmers which should also be considered during interpreting this association. The presence of *mcr-1* gene in the *E. coli* population in the day-old broiler chicks raises questions about the origin of the colistin resistance strains in such early age of birds. Vertical transmission of colistin resistance *E. coli* from broiler breeders to their offspring could be one explanation for the presence of *mcr-1* gene in the day-old chicks as indicated by previous studies^34,35^.

The pangenome analysis of 32 *mcr-1*-bearing commensal *E. coli* strains showed a highly flexible accessory genome. The *E. coli* core genome of this study is four times smaller than the pangenome. Previous studies have also shown a smaller size core genome of *E. coli*^36–38^. However, the core genome size is a comparative measurement because the core would be reduced when more genomes are added to the comparison^39^.

Almost all the isolates (91%) harboured at least one and several isolates harboured multiple virulence genes. The most frequent virulence gene was EAST-1 heat-stable toxin (*astA*) and the increased serum survival (*iss*) was the second most frequent virulence marker among the commensal strains. The *astA* gene is commonly found in enterotoxigenic *E. coli* (ETEC)^40^. ETEC is one of the leading causes of severe diarrhoea in young children in developing countries like Bangladesh^41^. Furthermore, increased serum survival gene *iss* is a well-known virulence marker of extraintestinal pathogenic *E. coli* (ExPEC) in avian species^42^, however, the presence of this gene was not found in combination with other virulence markers of avian pathogenic *E. coli* (APEC). Additionally, the dominant ST type among the isolates was ST43 (n = 6). This type is often found in human clinical infections as carbapenemase-producing *E. coli*^43,44^. The presence of virulence markers in the commensal *E. coli* strains isolated from poultry that have genetic similarity with diverse pathogenic clones indicates their potential of transferring virulence genes to pathogenic clones of *E. coli* in humans and animals.

Our study revealed that all the *mcr-1*-positive *E. coli* isolates carry multiple ARGs. The presence of tetracycline resistance genes in more than 70% of the *mcr-1*-bearing isolates indicated an alarming spread of tetracycline resistance genes among the commensal population. Although tetracycline is less commonly used in broiler production in Bangladesh^9^, its increased resistance in commensal *E. coli* could be an indication of resistance selection by a bystander effect of other antimicrobials^45^. A previous study from Bangladesh has identified tetracycline resistance genes in multidrug resistance *E. coli* strains isolated from children^46^. Earlier studies identified a varied prevalence of tetracycline resistance genes in *E. coli* isolated from both humans and animals^47^. One of the strains harboured extended-spectrum β-lactamase-producing gene *bla*_CTX-M-55_*.* Co-harbouring of *mcr-1* and *bla*_CTX-M-55_ gene was previously reported in the pyelonephritis case in a three-year child in France, caused by *E. coli*^48^. A combination of a mutation in the *gyrA* gene and the *parC* gene was detected in several isolates suggesting resistance to fluroquinolones^49,50^. The presence of single mutations in the *gyrA* gene altering serine 83 to leucine (S83L) and the double mutations in the *gyrA* gene altering aspartic acid (D87N) and serine (S83L) is known to confer high resistance capability of *E. coli* strains to fluroquinolones^51^.

All the 32 isolates harboured multiple plasmid replicons. The abundant plasmid types such as ColRNAI, IncFIB, IncHI2, and IncI2 were previously shown as *mcr-1*-carrying plasmid replicon^52,53^. The *mcr-1* gene mobilizing transposon component IS*Apl1* was detected in all the isolates except two. Evidence shows that IS*Apl1* plays a pivotal role in the spread of *mcr-1* gene^54^. In some cases, one or both copies of IS*Apl1* can be lost, however, a single copy of upstream IS*Apl1* is capable of mobilizing *mcr-1* genes^55^.

Phylogenetic comparison of the strains identified in the present study with an international collection of colistin-resistant *E. coli* showed that the *mcr-1*-bearing *E. coli* strains of this study were highly diverse. Only two small clusters of the isolates from this study were observed; these encompassed strains of ST43 (n = 6) and ST4965 (n = 5), respectively. All other isolates were found to be diffusely distributed across the tree according to the SNP phylogeny. Similarly, a high level of phylogenetic diversity of *mcr-1*-bearing *E. coli* was reported previously^53^. The scattering of *mcr-1-*bearing *E. coli* into genetically diverse strains suggests that the transfer of the gene between strains is a frequent event in the gut of broilers in the poultry farms in Bangladesh.

This study has some limitations. First, the study was conducted in a short time frame which did not allow us to observe if there is any seasonal variation on the AMR and genetic diversity of *E. coli*. Second, the study was conducted in a certain geographical region in Bangladesh. The addition of more farms from different regions of the country would bring a more detailed picture of the *E. coli* diversity. Third, only broiler chickens were investigated in this study, whereas layer and backyard chickens are also a major part of poultry production in Bangladesh. Finally, although the study identified the *mcr-1* gene in *E. coli* from day-old chicks, however, it was not possible to clarify whether the source of the resistant-strains was a vertical transmission from breeder flocks or not.

In conclusion, this first population level study on commensal *E. coli* and colistin resistance in broiler chicken encompassing different stages of production has revealed a high genetic diversity of commensal *E. coli* in broiler chicken in Bangladesh. The study also revealed a high frequency of *mcr-1* carriage among the commensal *E. coli* population and showed that the carriage was positively associated with colistin use in chicken production. The presence of *mcr-1* gene in a diverse *E. coli* population suggests a massive horizontal spread of *mcr-1* gene rather than clonal expansion. These results call for immediate action from the policy makers to stop imprudent use or to actively regulate the rational use of colistin along with other critically important antimicrobials in poultry production in Bangladesh.

## Materials and methods

### Study farms and sampling

A longitudinal study was conducted between August to September 2018. Commercial broiler chicken farms in the Chattogram division of Bangladesh was chosen for this study. Broiler farm owners were invited to participate in the study. Farms were selected based on the starting date of a new flock during the study period, minimum flock size of 500 birds, and geographically well-representing across the division. Pooled faecal samples from each farm were collected longitudinally at three sampling times: at day1, day15, and day28 of the production. The pooled sample consists of five randomly picked fresh faecal droplets collected from four corners and the centre of the flock. A trained veterinarian collected the samples and brought them to the laboratory on the same day with a proper transporting system in a cooling box. A questionnaire survey was also conducted for epidemiological data on each sampling time. The parameters investigated includes strains of birds, use of antimicrobials, sources of food and water, and farm biosecurity practices. All farm owners were agreed to participate in the study and informed consent was taken before sampling from farms and questionnaire survey on farming data. The animal ethical committee of Chattogram Veterinary and Animal Sciences University (CVASU), Bangladesh approved the study protocol and collection of animal related data by a questionnaire.

### Bacterial isolation and identification

*E. coli* were isolated and quantified from the faecal samples by drop plate method^56^ on MacConkey agar (MCA) (Oxoid, United Kingdom). Lactose-positive, red, non-mucoid colonies were randomly selected from each sample and sub cultured onto Eosin Methylene Blue agar (EMB) (Scharlau, Spain) for biochemical confirmation. Species identity of *E. coli* was confirmed by standard biochemical properties followed by species-specific multiplex PCR^57^. Primers for the *uidA* gene and flanking region of the *uspA* gene were used. The amplified PCR products were separated by electrophoresis at 70 V in a 2% agarose gel (Sigma-Aldrich, USA) containing ethidium bromide (AMRESCO, USA) followed by visualizing under UV light. Gene Ruler 100 bp Plus DNA Ladder (Thermo Fisher Scientific, USA) was used to standardize the PCR band images. Confirmed *E. coli* isolates were preserved at -80 ˚C after subculture onto the Blood agar plate (BA) (Oxoid, UK).

### PCR for *mcr* genes and minimum inhibitory concentration (MIC) determination

All isolates were screened by a multiplex PCR to detect five *mcr* genes (*mcr-1* to *mcr-5*) as described previously^58^. *E. coli* NCTC 13846, *E. coli* KP37^11^, *E. coli* 2013-SQ352^58^*, E. coli* DH5α^13^, and *Salmonella* 13-SA01718^14^ were used as the positive controls for *mcr-1*, *mcr-2*, *mcr-3*, *mcr-4*, and *mcr-5*, respectively. Gene Ruler 100 bp Plus DNA Ladder (Thermo Fisher Scientific, USA) was used as an external reference control. All the *mcr*-positive *E. coli* strains were subjected to MIC determination. The MIC for colistin was determined by the broth microdilution (BMD) method according to ISO-standard (20776-1)^59^. Colistin sulphate (Sigma-Aldrich, Saint Louis, MO, USA) and cation-adjusted Mueller–Hinton Broth II (Sigma-Aldrich, St Louis, MO, USA) were used in the BMD test. The quality control of the experiment was monitored by a resistant strain of *E. coli* NCTC 13846 (*mcr-1*-positive) and a susceptible strain of *E. coli* ATCC 25922. To test the colistin susceptibility of the *mcr-1*-negative isolates, all the isolates were streaked on the MacConkey agar with 2 mg/L colistin sulphate. Growth was scored after overnight incubation of agar plates at 37˚C. The *mcr-1*-negative isolates which showed growth at this concentration were subjected to MIC determination for colistin as above.

### Repetitive extragenic palindromic-PCR (REP-PCR) fingerprinting

The genetic diversity of the *E. coli* isolates was determined by REP-PCR. The primers used for REP-PCR fingerprinting were Rep1R-I (5′-III ICG ICG ICA TCI GGC-3′) and Rep2-I (5′-ICG ICT TAT CIG GCC TAC-3′). The PCR was performed according to a previously described protocol^60^. *E. coli* ATCC 25922 and sterile MilliQ water were used as positive and negative controls, respectively. As an external reference control, GeneRuler 100 bp plus DNA ladder (Thermo Fisher Scientific, USA) was used to standardize the fingerprint profiles. GelJ, an image analyzing program, was used to analyze REP-PCR DNA fingerprints data^61^. The normalization of every gel images was done by 100 bp plus DNA ladder (Thermo Fisher Scientific, USA) as an external reference. Unique REP-type was assigned using the Dice similarity method with more than 90% band similarity and 4% tolerance. The REP-PCR-based genetic diversity of commensal *E. coli* strains as well as the diversity of colistin resistant *E. coli* strains were determined by the Shannon diversity index (*H´*). The following formula^62^ was used to calculate the diversity-

$$H^{{\prime }} = - \mathop\sum \limits_{i = 1}^{s} p_{i} \ln p_{i}$$

S denotes the number of unique genotype and *p*_*i*_ is the number of isolates sharing the same genotype [i] over the total number of isolates.

### Whole genome sequencing (WGS) and analysis

A total of 32 *mcr-1*-bearing *E. coli* isolates among the dominant REP-types (REP-type with at least three isolates) were selected for WGS. The genomic DNA was extracted using the DNeasy Blood and Tissue Kit (Qiagen, Hilden, Germany). The sequencing library was prepared according to the Illumina protocol and paired-end next generation sequencing was performed on the Illumina MiSeq platform (Illumina, San Diego, USA).

#### Quality checking and de-novo assembly of sequencing reads

The quality of the sequencing reads was tested using fastqc (Galaxy Version 0.72 + galaxy1)^63^ in the Galaxy platform^64^. We used “Galaxy Europe Instance” for all other downstream analyses related to the Galaxy platform. The raw reads that passed quality control were *de-novo* assembled into a draft genome using a hybrid assembly pipeline Unicycler (Galaxy Version 0.4.7.0)^65^. The pipeline uses both Illumina reads and long reads to produce complete and accurate assemblies. We used default assembly parameters. Genome assembly quality was assessed using Quast (Galaxy Version 5.0.2 + galaxy0)^66^.

#### Genome annotation and pangenome analysis

The *de-novo* assembled genomes were annotated using the prokaryotic genome annotation pipeline (PROKKA, Galaxy Version 1.13)^67^. Core and accessory genome comparison of the 32 isolates isolated from of this current study was performed using the Roary pangenome pipeline (Galaxy Version 3.10.2)^68^. Roary generates a core gene alignment from gff3 files produced by PROKKA annotation. A 99% identity cut-off was used to define the “core” gene among the isolates. A concatenated core gene alignment of each of the core genes of all the isolates was generated. The combined core gene alignment was used to construct a maximum-likelihood phylogenetic tree using RAxML (Galaxy Version 1.0.0)^69^. We used the default 1000 fast bootstraps on the best likelihood tree constructed with the General Time Reversible (GTR) substitution model and with a Gamma rate of correction heterogeneity. The gene presence/absence file generated by the Roary pangenome annotation pipeline and the core gene phylogenetic tree were visualized using a web based interactive visualization tool Phandango^70^.

#### Global phylogeny

For the phylogenetic comparison of our isolates at global context, we included WGS data of 126 *mcr-1*-bearing *E. coli* strains listed in a previous study (Supplementary File 1)^71^. The global isolates those have complete metadata were considered for comparison with the *mcr-1*-positive isolates obtained in this study. A global scale phylogenetic comparison was conducted with all the 158 isolates^71^. Galaxy-based Snippy^72^ tool (Galaxy Version 4.3.6 + galaxy2) was used to detect SNPs between a reference genome (*E. coli* K12 MG1665, accession NC_000913.3) and the sequencing reads of interest. Multiple Snippy outputs were combined into a “core SNPs” alignment using Snippy Core (Galaxy Version 4.3.6). The “core site” represents a common genomic position present in all the genomes. The “core SNPs” alignment was used to build a high-resolution phylogeny (ignoring possible recombination). We reconstructed a maximum likelihood phylogenetic tree using FastTree^73^ (Galaxy Version 2.1.10) with GTR + CAT Nucleotide evolution model. The phylogenetic tree was visualized using an interactive webtool iTOL^74^.

#### In silico* typing*

Acquired antimicrobial resistance genes and chromosomal mutations were detected using Resfinder version 3.2 on Center for Genomic Epidemiology (CGE) web server^75^. CGE webserver was used for further molecular typing of the isolates. Multi-locus sequence typing (MLST), serotyping, virulence determination, plasmid replicon identification and typing were performed using MLST version 2.0, SerotypeFinder version 2.0, VirulenceFinder version 2.0, PlasmidFinder version 2.0 and pMLST version 2.0, respectively^76–79^. The presence of the insertion sequence IS*Apl1* belonged to the IS*30* family of transposons was identified using the ISfinder online tool^80^.

### Statistical analysis

Epidemiological data were analyzed in *R 3.5.1*^81^. Univariable analysis followed by multivariable logistic regression analysis was performed to identify possible risk factors associated with the prevalence of the *mcr-1* gene in the *E. coli* population. First, we used the univariable logistic regression analysis to identify potential risk factors to be included in the multivariable analysis. Variables with a *p*-value of less than 0.1 in the univariable analysis were selected for multivariable analysis. Due to the hierarchical data structure, we used three level logistic regression model. The individual observation (1200 *E. coli* isolates) were nested within three sampling times (day1, day15, and day28) at level-2 which were in turn nested within twenty farms at level-3. The R package lme4^82^ was used for logistic regression analysis.

### Ethical approval

The study protocol and a questionnaire to collect animal related data were developed in accordance with relevant guidelines and regulations in Bangladesh which was approved by the animal ethical committee of Chattogram Veterinary and Animal Sciences University (CVASU), Bangladesh (Approval No. CVASU/Dir(R&E)EC/-2019/39(2/6)). Informed consent was taken from all participatory farm owners before sampling and data collection from farms. No animals were handled or harmed in this study as only fecal droppings were collected from the farm floor.

## Supplementary information

Supplementary Information 1.

Supplementary Information 2.

Supplementary Tables.

## Data Availability

The genome sequencing data were submitted to the European Nucleotide Archive (ENA) under the project accession number PRJEB34000. All other data generated or analysed in this study are included in the manuscript and in the Supplementary Materials.
