# Supplementary material for: High prevalence of mcr-1-encoded colistin resistance in commensal Escherichia coli from broiler chicken in Bangladesh
Source: Sci Rep. 2020 Oct 29;10:18637. doi: 10.1038/s41598-020-75608-2 (PMC7596488; doi:10.1038/s41598-020-75608-2)
Supplement: Supplementary file 3 — Supplementary Tables. [file 41598_2020_75608_MOESM3_ESM.docx]

**Supplementary Materials for**

**Title: High prevalence of *mcr-1*-encoded colistin resistance in commensal *Escherichia coli* from broiler chicken in Bangladesh**

Shahana Ahmed^1^, Tridip Das^2^, Md Zohorul Islam^1,2,3,4^, Ana Herrero-Fresno^1^, Paritosh Kumar Biswas^2^, John Elmerdahl Olsen^1#^

^1^Department of Veterinary and Animal Sciences, Faculty of Health and Medical Sciences, University of Copenhagen, Frederiksberg, Denmark

^2^Department of Microbiology and Veterinary Public Health, Chattogram Veterinary and Animal Sciences University, Bangladesh

^3^Section on Pathophysiology and Molecular Pharmacology, Joslin Diabetes Center, Boston, MA, USA

^4^Department of Microbiology and Immunobiology, Harvard Medical School, Boston, MA, USA

Shahana Ahmed: shahana@sund.ku.dk, Tridip Das: das.vet671@gmail.com, Md Zohorul Islam: MDZohorul.islam@joslin.harvard.edu/zois@sund.ku.dk, Ana Herrero-Fresno: ahefr@sund.ku.dk, Paritosh Kumar Biswas: biswaspk2000@yahoo.com, John Elmerdahl Olsen: [jeo@sund.ku.dk](mailto:jeo@sund.ku.dk)

^#^Corresponding author, John Elmerdahl Olsen; Email: jeo@sund.ku.dk

**Supplementary File 1** (Uploaded as separate Microsoft excel file). List of strains and metadata used for phylogenetic comparison.

**Supplementary File 2** (Uploaded as separate Microsoft excel file). Genome annotation data of 32 *mcr-1*-positive commensal *E. coli*.

**Supplementary Table 1.** Farm characteristics and history of antimicrobial usages

| Farm | Farm size (No. bird) | Broiler strains | Use of antimicrobials | Source of prescribed antimicrobials |
| --- | --- | --- | --- | --- |
| F01 | 1120 | Cobb500 | Ciprofloxacin, Doxycycline, Neomycin, Sulpha drugs | Veterinarians |
| F02 | 4000 | Cobb500 | Amoxicillin, Colistin, Oxytetracycline, Sulpha drugs | Veterinarians |
| F03 | 1600 | Cobb500 | Colistin, Doxycycline, Oxytetracycline, Sulpha drugs | Veterinarians |
| F04 | 1000 | Cobb500 | Amoxicillin, Colistin | Veterinarians |
| F05 | 920 | Cobb500 | Amoxicillin, Enrofloxacin, Oxytetracycline, Sulpha drugs | Veterinarians |
| F06 | 1700 | Cobb500 | Amoxicillin, Ciprofloxacin, Colistin, Oxytetracycline, Sulpha drugs | Veterinarians |
| F07 | 900 | Cobb500 | Amoxicillin, Ciprofloxacin, Colistin, Flumequine, Oxytetracycline | Veterinarians |
| F08 | 2100 | Cobb500 | Amoxicillin, Colistin, Sulpha drugs | Veterinarians |
| F09 | 800 | Cobb500 | Amoxicillin, Ciprofloxacin | Veterinarians |
| F10 | 800 | Cobb500 | Amoxicillin, Colistin, Sulpha drugs | Veterinarians |
| F11 | 1300 | Cobb500 | Amoxicillin, Ciprofloxacin, Colistin, Oxytetracycline, Sulpha drugs | Veterinarians |
| F12 | 1300 | Ross-308 | Unrecognised | Self-medication |
| F13 | 1200 | Lohmann | Colistin, Oxytetracycline | Self-medication |
| F14 | 1200 | Ross-308 | Colistin, Sulpha drugs | Self-medication |
| F15 | 800 | Cobb500 | Colistin, Pefloxacin, Sulpha drugs | Veterinarians |
| F16 | 1500 | Cobb500 | Cefalexin, Ceftriaxone, Colistin, Sulpha drugs | Veterinarians |
| F17 | 800 | Cobb500 | Enrofloxacin | Veterinarians |
| F18 | 840 | Cobb500 | Amoxicillin, Colistin, Enrofloxacin, Norfloxacin, Oxytetracycline | Veterinarians |
| F19 | 800 | Cobb500 | No antibiotic use | - |
| F20 | 800 | Cobb500 | Ciprofloxacin, Enrofloxacin, Sulpha drugs | Veterinarians |

**Supplementary Table 2**. Distribution of REP-types and *mcr-1*-bearing *E. coli* across the 20 broiler farms

| REP-type | No.  *E. coli* | No. *mcr-1-*bearing  *E. coli* | Farm (No. *E. coli*) |
| --- | --- | --- | --- |
| R056 | 32 | 11 | F02(1), F04(2), F06(1), F07(1), F08(3), F09(5), F10(4), F11(1), F12(3), F13(3), F14(1), F16(1), F17(6) |
| R029 | 31 | 11 | F01(1), F02(7), F03(6), F04(8), F05(2), F06(1), F07(1), F08(1), F09(1), F14(1), F16(1), F20(1) |
| R054 | 25 | 6 | F02(2), F05(1), F06(1), F07(8), F09(1), F12(2), F13(1), F14(1), F17(2), F18(2), F20(4) |
| R046 | 24 | 16 | F02(2), F04(12), F05(3), F06(1), F07(1), F11(1), F13(2), F14(1), F17(1) |
| R040 | 22 | 3 | F02(1), F04(12), F05(6), F06(2), F20(1) |
| R051 | 19 | 11 | F02(2), F03(10), F05(2), F06(1), F12(1), F14(1), F18(1), F19(1) |
| R117 | 19 | 4 | F05(1), F08(1), F10(3), F13(1), F14(8), F16(1), F17(1), F18(1), F19(1), F20(1) |
| R096 | 17 | 6 | F04(2), F05(1), F09(7), F11(1), F12(1), F13(1), F14(1), F15(1), F16(1), F20(1) |
| R230 | 17 | 6 | F10(2), F11(3), F12(1), F14(5), F15(1), F16(1), F18(2), F19(1), F20(1) |
| R255 | 17 | - | F12(17) |
| R045 | 16 | 3 | F02(1), F06(1), F07(1), F10(1), F13(2), F15(2), F17(2), F18(2), F19(2), F20(2) |
| R148 | 16 | 4 | F07(1), F08(2), F09(9), F11(1), F12(1), F17(1), F18(1) |
| R052 | 15 | 5 | F02(1), F06(1), F08(1), F12(1), F13(1), F14(1), F15(3), F16(1), F18(3), F19(2) |
| R066 | 15 | 3 | F02(1), F05(4), F06(4), F10(2), F13(1), F15(1), F16(1), F19(1) |
| R067 | 15 | 2 | F03(4), F06(1), F10(1), F11(2), F19(2), F20(5) |
| R114 | 15 | - | F05(2), F08(1), F10(2), F15(1), F17(1), F18(2), F19(5), F20(1) |
| R116 | 15 | 4 | F05(1), F06(2), F08(1), F11(2), F13(2), F14(1), F15(1), F17(1), F18(3), F20(1) |
| R032 | 14 | 2 | F02(5), F05(2), F06(4), F14(1), F17(2) |
| R057 | 13 | - | F02(1), F03(1), F05(1), F07(6), F11(1), F17(1), F18(1), F20(1) |
| R081 | 13 | 6 | F03(1), F06(2), F08(5), F09(1), F10(2), F15(2), F15(3), F16(1), F18(3), F19(2) |
| R044 | 12 | - | F02(3), F07(5), F15(1), F18(1), F19(2) |
| R152 | 12 | - | F07(9), F16(1), F18(2) |
| R173 | 11 | 4 | F08(3), F11(1), F14(3), F15(3), F18(1) |
| R079 | 10 | 8 | F03(6), F12(1), 148(1), F18(1), F19(1) |
| R093 | 10 | - | F04(4), F06(1), F11(2), F13(2), F18(1) |
| R113 | 10 | 2 | F05(3), F11(2), F12(2), F13(1), F14(1), F16(1) |
| R324 | 10 | 10 | F16(10) |
| R039 | 9 | - | F02(2), F03(1), F04(1), F08(2), F18(1), F19(2) |
| R068 | 9 | 3 | F03(3), F04(2), F06(1), F10(2), F15(1) |
| R149 | 9 | 6 | F07(1), F09(2), F10(3), F13(1), F18(2) |
| R216 | 9 | 4 | F10(1), F11(1), F12(1), F13(2), F14(2), F15(1), F19(1) |
| R241 | 9 | - | F11(3), F20(6) |
| R003 | 8 | - | F01(8) |
| R031 | 8 | - | F01(2), F02(2), F04(3), F8(1) |
| R055 | 8 | 2 | F02(3), F04(1), F11(1), F13(1), F18(2) |
| R107 | 8 | 2 | F05(1), F17(4), F18(2), F19(1) |
| R142 | 8 | - | F07(3), F11(4), F18(1) |
| R207 | 8 | 2 | F10(2), F15(2), F17(2), F18(2) |
| R270 | 8 | - | F13(7), F15(1) |
| R352 | 8 | 5 | F19(8) |
| R365 | 8 | - | F20(8) |
| R027 | 7 | 3 | F01(1), F14(2), F15(2), F19(1), F20(1) |
| R063 | 7 | - | F02(1), F06(2), F08(2), F10(1), F11(1) |
| R069 | 7 | - | F03(1), F14(1), F14(4), F20(1) |
| R147 | 7 | - | F07(2), F08(3), F15(1), F16(1) |
| R224 | 7 | - | F10(1), F16(1), F19(5) |
| R242 | 7 | 2 | F11(1), F12(4), F16(1), F19(1) |
| R269 | 7 | - | F13(7) |
| R291 | 7 | 2 | F14(1), F15(5), F20(1) |
| R337 | 7 | - | F17(7) |
| R009 | 6 | - | F01(6) |
| R041 | 6 | - | F02(1), F04(2), F13(1), F15(1), F17(1) |
| R043 | 6 | - | F02(5), F19(1) |
| R064 | 6 | - | F02(1), F05(2), F13(2), F19(1) |
| R082 | 6 | 6 | F03(6) |
| R090 | 6 | - | F04(1), F11(2), F14(1), F19(1), F20(1) |
| R094 | 6 | - | F04(3), F14(1), F20(2) |
| R100 | 6 | - | F05(5), F14(1) |
| R155 | 6 | - | F07(2), F11(2), F15(1), F19(1) |
| R288 | 6 | - | F14(2), F16(4) |
| R095 | 5 | 2 | F04(1), F12(1), F14(2), F17(1) |
| R129 | 5 | 2 | F06(1), F13(1), F18(1), F20(2) |
| R136 | 5 | - | F06(3), F11(1), F12(1) |
| R171 | 5 | - | F08(2), F09(1), F13(1), F15(1) |
| R195 | 5 | 4 | F09(4), F20(1) |
| R203 | 5 | 2 | F09(1), F11(2), F13(1), F14(1) |
| R232 | 5 | 3 | F10(1), F16(1), F17(2), F18(1) |
| R274 | 5 | 4 | F13(1), F14(4) |
| R012 | 4 | - | F01(4) |
| R014 | 4 | - | F01(4) |
| R017 | 4 | - | F01(4) |
| R030 | 4 | - | F01(1), F15(2), F16(1) |
| R034 | 4 | - | F02(1), F05(1), F06(2) |
| R036 | 4 | - | F01(1), F13(1), F15(1), F17(1) |
| R048 | 4 | - | F02(1), F06(1), F17(1), F19(1) |
| R078 | 4 | - | F03(1), F09(1), F12(1), F13(1) |
| R080 | 4 | 4 | F03(4) |
| R102 | 4 | - | F05(1), F17(2), F20(1) |
| R121 | 4 | 3 | F06(1), F10(1), F16(2) |
| R127 | 4 | 3 | F06(2), F11(1), F18(1) |
| R144 | 4 | - | F07(4) |
| R165 | 4 | 2 | F08(1), F12(1), F18(2) |
| R179 | 4 | - | F08(2), F12(2) |
| R194 | 4 | - | F19(4) |
| R211 | 4 | - | F10(4) |
| R217 | 4 | 2 | F10(1), F15(1), F16(1), F20(1) |
| R239 | 4 | - | F11(2), F16(2) |
| R260 | 4 | - | F12(1), F16(1), F18(2) |
| R262 | 4 | - | F12(1), F18(2), F19(1) |
| R265 | 4 | 2 | F12(1), F13(2), F15(1) |
| R287 | 4 | - | F14(4) |
| R323 | 4 | 4 | F16(4) |
| R037 | 3 | - | F02(1), F15(1), F18(1) |
| R060 | 3 | - | F02(1), F14(1), F18(1) |
| R062 | 3 | - | F02(1), F16(1), F18(1) |
| R099 | 3 | - | F05(1), F07(2) |
| R108 | 3 | 2 | F05(1), F19(1), F20(1) |
| R110 | 3 | 3 | F05(3) |
| R119 | 3 | - | F06(1), F09(1), F12(1) |
| R125 | 3 | - | F06(1), F11(2) |
| R141 | 3 | - | F06(1), F12(1), F14(1) |
| R163 | 3 | - | F08(3) |
| R169 | 3 | - | F08(1), F18(2) |
| R170 | 3 | - | F08(2), F15(1) |
| R175 | 3 | - | F08(3) |
| R184 | 3 | 2 | F08(2), F15(1) |
| R196 | 3 | - | F09(1), F10(2) |
| R205 | 3 | - | F09(3) |
| R214 | 3 | - | F10(3) |
| R218 | 3 | - | F10(1), F12(2) |
| R244 | 3 | 2 | F11(1), F13(1), F20(1) |
| R251 | 3 | - | F11(1), F16(1), F17(1) |
| R273 | 3 | - | F04(1), F13(1), F19(1) |
| R316 | 3 | - | F16(3) |
| R001 | 2 | - | F01(2) |
| R002 | 2 | - | F01(2) |
| R005 | 2 | - | F01(2) |
| R006 | 2 | - | F01(2) |
| R008 | 2 | - | F01(2) |
| R013 | 2 | - | F01(2) |
| R019 | 2 | - | F01(2) |
| R028 | 2 | - | F01(1), F03(1) |
| R035 | 2 | - | F02(1), F05(1) |
| R042 | 2 | - | F02(1), F03(1) |
| R047 | 2 | - | F02(1), F20(1) |
| R059 | 2 | - | F02(1), F11(1) |
| R061 | 2 | - | F02(1), F06(1) |
| R072 | 2 | - | F03(1), F04(1) |
| R074 | 2 | - | F03(1), F19(1) |
| R075 | 2 | - | F03(1), F19(1) |
| R076 | 2 | - | F03(1), F17(1) |
| R083 | 2 | - | F03(1), F08(1) |
| R084 | 2 | - | F03(2) |
| R097 | 2 | - | F05(2) |
| R101 | 2 | - | F05(2) |
| R104 | 2 | - | F05(1), F18(1) |
| R111 | 2 | - | F05(2) |
| R112 | 2 | - | F05(2) |
| R128 | 2 | 2 | F06(2) |
| R130 | 2 | 2 | F06(2) |
| R132 | 2 | - | F06(2) |
| R139 | 2 | - | F06(1), F09(1) |
| R145 | 2 | - | F07(1), F12(1) |
| R150 | 2 | - | F07(1), F16(1) |
| R153 | 2 | - | F07(1), F12(1) |
| R154 | 2 | - | F07(1), F09(1) |
| R159 | 2 | 2 | F07(2) |
| R162 | 2 | - | F08(1), F16(1) |
| R168 | 2 | - | F08(1), F18(1) |
| R180 | 2 | - | F08(1), F19(1) |
| R188 | 2 | - | F09(2) |
| R200 | 2 | - | F09(1), F15(1) |
| R210 | 2 | - | F10(2) |
| R215 | 2 | - | F10(1), F18(1) |
| R221 | 2 | - | F10(2) |
| R231 | 2 | - | F10(1), F13(1) |
| R233 | 2 | - | F10(1), F16(1) |
| R235 | 2 | - | F11(1), F13(1) |
| R243 | 2 | - | F11(1), F15(1) |
| R248 | 2 | - | F11(1), F15(1) |
| R254 | 2 | - | F11(2) |
| R286 | 2 | - | F14(1), F16(1) |
| R295 | 2 | - | F14(1), F19(1) |
| R296 | 2 | - | F15(2) |
| R304 | 2 | - | F15(1), F19(1) |
| R308 | 2 | - | F15(1), F20(1) |
| R312 | 2 | - | F16(1), F18(1) |
| R326 | 2 | 2 | F16(2) |
| R328 | 2 | - | F17(1), F20(1) |
| R345 | 2 | - | F18(2) |
| R346 | 2 | - | F18(2) |
| R353 | 2 | - | F19(2) |
| R354 | 2 | - | F19(2) |
| R363 | 2 | - | F20(2) |
| Singleton | 193* | 85** | F01(14), F02(7), F03(7), F04(4), F05(6), F06(13), F07(7), F08(14), F09(13), F10(13), F11(12), F12(10), F13(10), F14(7), F15(13), F16(10), F17(14), F18(4), F19(6), F20(9) |

*Singletons: R004, R007, R010, R011, R015, R016, R018, R020, R021, R022, R023, R024, R025, R026, R033, R038, R049, R050, R053, R058, R065, R070, R071, R073, R077, R085, R086, R087, R088, R089, R091, R092, R098, R103, R105, R106, R109, R115, R118, R120, R122, R123, R124, R126, R131, R133, R134, R135, R137, R138, R140, R143, R146, R151, R156, R157, R158, R160, R161, R164, R166, R167, R172, R174, R176, R177, R178, R181, R182, R183, R185, R186, R187, R189, R190, R191, R192, R193, R197, R198, R199, R201, R202, R204, R206, R208, R209, R212, R213, R219, R220, R222, R223, R225, R226, R227, R228, R229, R234, R236, R237, R238, R240, R245, R246, R247, R249, R250, R252, R253, R256, R257, R258, R259, R261, R263, R264, R266, R267, R268, R271, R272, R275, R276, R277, R279, R280, R281, R282, R283, R284, R285, R289, R290, R292, R293, R294, R297, R298, R299, R300, R301, R302, R303, R305, R306, R307, R309, R310, R311, R313, R314, R315, R317, R318, R319, R320, R321, R322, R325, R327, R329, R330, R331, R332, R333, R334, R335, R336, R338, R339, R340, R341, R342, R343, R344, R347, R348, R349, R350, R351, R355, R356, R357, R358, R359, R360, R361, R362, R364, R366, R367, R368;

***mcr-1*-bearing singletons: R002, R017, R018, R030, R031, R035, R036, R037, R039, R043, R044, R047, R049, R050, R057, R060, R062, R063, R085, R087, R090, R093, R100, R102, R114, R118, R126, R132, R139, R141, R145, R152, R153, R155, R156, R158, R170, R171, R179, R185, R186, R190, R191, R192, R193, R194, R200, R218, R222, R225, R228, R235

R243, R246, R248, R256, R258, R260, R267, R268, R273, R275, R281, R282, R283, R284, R285, R286, R295, R303, R304, R305, R306, R307, R308, R309, R310, R313, R322, R339, R351, R363, R364, R365, R366.

**Supplementary Table 3.** Annotation summery of 32 commensal *E. coli* genome identified by PROKKA

| Isolate/variable | Contigs | Bases | tRNA | rRNA | CDS | tmRNA | Repeat region |
| --- | --- | --- | --- | --- | --- | --- | --- |
| S001 | 412 | 5088472 | 81 | 4 | 4861 | 1 | 2 |
| S002 | 264 | 5050521 | 76 | 3 | 4727 | 1 | 2 |
| S003 | 475 | 5002614 | 81 | 4 | 4734 | 1 | 2 |
| S004 | 213 | 4458141 | 83 | 5 | 4102 | 1 | 1 |
| S005 | 421 | 4999934 | 76 | 6 | 4750 | 1 | 0 |
| S024 | 196 | 4901320 | 82 | 3 | 4634 | 1 | 2 |
| S025 | 352 | 5258027 | 85 | 4 | 5008 | 1 | 2 |
| S026 | 345 | 5177832 | 81 | 3 | 4868 | 1 | 1 |
| S027 | 290 | 5168466 | 79 | 3 | 4870 | 1 | 1 |
| S028 | 310 | 5132795 | 85 | 3 | 4873 | 1 | 1 |
| S029 | 310 | 5449133 | 79 | 4 | 5065 | 1 | 2 |
| S030 | 265 | 5012124 | 81 | 3 | 4731 | 1 | 2 |
| S031 | 450 | 5416921 | 79 | 4 | 5227 | 1 | 2 |
| S032 | 530 | 5097772 | 78 | 3 | 4916 | 1 | 2 |
| S033 | 405 | 5085732 | 82 | 4 | 4842 | 1 | 1 |
| S034 | 346 | 5232367 | 84 | 4 | 4989 | 1 | 1 |
| S035 | 193 | 4883973 | 79 | 4 | 4602 | 1 | 2 |
| S036 | 664 | 5020016 | 75 | 4 | 4720 | 1 | 2 |
| S037 | 219 | 4983708 | 84 | 3 | 4726 | 1 | 2 |
| S038 | 276 | 5153909 | 86 | 3 | 4922 | 1 | 2 |
| S039 | 259 | 4846914 | 78 | 3 | 4491 | 1 | 2 |
| S040 | 184 | 5149330 | 84 | 3 | 4817 | 1 | 2 |
| S041 | 415 | 4994990 | 76 | 5 | 4747 | 0 | 1 |
| S042 | 202 | 4826248 | 74 | 3 | 4495 | 1 | 2 |
| S043 | 392 | 5127851 | 83 | 4 | 4882 | 1 | 2 |
| S044 | 405 | 5040593 | 80 | 4 | 4807 | 1 | 2 |
| S045 | 289 | 5331089 | 86 | 4 | 5090 | 1 | 2 |
| S046 | 236 | 5258164 | 83 | 5 | 4994 | 1 | 2 |
| S047 | 151 | 5195606 | 79 | 4 | 4821 | 1 | 1 |
| S048 | 343 | 5108986 | 78 | 6 | 4878 | 0 | 1 |
| S049 | 177 | 5074654 | 78 | 4 | 4768 | 1 | 2 |
| S050 | 183 | 5039722 | 79 | 2 | 4727 | 1 | 2 |
| Average | 318 | 5080248 | 80 | 4 | 4803 | 0.94 | 1.7 |
| SD | 117.46 | 185246 | 3.3 | 0.91 | 204.4 | 0.23 | 0.55 |
| median | 300 | 5087102 | 80.5 | 4 | 4819 | 1 | 2 |
| minimum | 151 | 4458141 | 74 | 2 | 4102 | 0 | 0 |
| maximum | 664 | 5449133 | 86 | 6 | 5227 | 1 | 2 |

**Supplementary Table 4.** Genomic features of commensal *E. coli* isolates

| **ID** | **REP-type** | **MLST type** | **Plasmids** | **Virulence genes** | **Serotypes** | **No. IS*Apl1*** |
| --- | --- | --- | --- | --- | --- | --- |
| S001 | R051 | ST-4965 | IncHI2, IncFII(pHN7A8), IncHI2A, IncI1, IncFIB(AP001918), IncN, ColRNAI | *astA* | O13:H30 | 2 |
| S002 | R029 | ST-3107 | IncFIB(pLF82), IncFIC(FII), IncI1, IncFIB(AP001918), IncFII(pCoo), ColE10, ColRNAI, Col(MG828) | *cba, iroN, iss, mchF, tsh* | O-:H12 | 2 |
| S003 | R046 | ST-4965 | IncHI2A, IncHI2, IncFIB(AP001918), IncFII(pHN7A8), IncN, ColRNAI | *astA* | O13:H30 | 2 |
| S004 | R056 | Unknown ST | IncX1, ColpVC, ColRNAI | *-* | O-:H34 | 0 |
| S005 | R324 | ST-1818 | IncFII(pSE11), IncHI2, IncHI2A, IncFII, IncFIB(pENTAS01), IncN, IncX1, ColE10, ColpVC, ColRNAI | *etpD, lpfA* | O116:H16 | 2 |
| S024 | R079 | ST-189 | IncFII, IncX1, ColpVC, ColRNAI | *astA* | O86:H21 | 2 |
| S025 | R054 | ST-43 | IncHI2A, IncHI2, IncFIB(AP001918), IncN, IncI2, ColRNAI | *iss* | O6:H10 | 2 |
| S026 | R081 | ST-48 | IncFIB(pLF82), IncHI2, IncHI2A, IncX1, p0111, ColRNAI | *-* | O-:H21 | 3 |
| S027 | R082 | ST-3107 | IncFIB(pLF82), IncI1, IncFIC(FII), IncFII(pCoo), IncFIB(AP001918), IncN, IncI2, IncX1, ColE10, Col(MG828), ColRNAI | *iroN, iss, mchF, tsh* | O98:H12 | 2 |
| S028 | R096 | ST-43 | IncI1, IncFIB(AP001918), Col(MGD2), IncI2 | *astA, iss* | O-:H10 | 2 |
| S029 | R149 | Unknown ST | IncHI2A, IncFII, IncHI2, IncFIB(AP001918), IncN, IncI2, p0111, ColRNAI | *air, astA, eilA, iha, ireA, iss* | O-:H16 | 2 |
| S030 | R230 | ST-43 | IncI1, IncFIB(AP001918), IncI2, Col(MGD2), ColRNAI | *astA, iss* | O6:H10 | 3 |
| S031 | R052 | ST-867 | IncI1, IncFIC(FII), IncHI1B(R27), IncFIA(HI1), IncFIB(AP001918), IncHI1A, IncI2, p0111, ColRNAI | *astA* | O13:H30 | 1 |
| S032 | R352 | Unknown ST | IncHI2A, IncHI2, IncFIB(AP001918), IncN, IncX1, ColE10, Col(MG828), ColRNAI | *-* | O27:H29 | 3 |
| S033 | R080 | ST-4965 | IncHI2A, IncHI2, IncFIB(AP001918), IncFII(pHN7A8), IncN, ColRNAI | *astA* | O13:H30 | 2 |
| S034 | R116 | ST-43 | IncHI2A, IncHI2, IncFIB(AP001918), IncN, IncX1, ColRNAI | *iss* | O6:H10 | 3 |
| S035 | R117 | ST-602 | IncFIC(FII), IncFIB(AP001918), IncX1, IncI2 | *cma, iroN, iss, lpfA* | O-:H21 | 1 |
| S036 | R148 | ST-867 | IncFIA(HI1), IncFIC(FII), IncFIB(AP001918), IncHI1B(R27), IncHI1A, IncI2, IncX1, Col3M, IncQ1, ColRNAI | *astA* | O-:H30 | 1 |
| S037 | R173 | ST-359 | IncFIC(FII), IncI1, IncFIB(AP001918), IncI2, ColRNAI | *cma, iroN, iss, lpfA* | O-:H32 | 0 |
| S038 | R195 | ST-43 | IncI1, IncFIB(AP001918), Col(MGD2), IncI2, ColpVC | *astA, iss* | O-:H10 | 2 |
| S039 | R216 | ST-206 | IncFIC(FII), IncFIB(AP001918), IncX1, ColRNAI | *astA, ireA* | O-:H51 | 3 |
| S040 | R274 | ST-1196 | IncFIB(AP001918), IncFII(pCoo), IncI2, IncX1, ColE10, IncX4, Col(MG828) | *iroN, iss, lpfA, mchF* | O76:H23 | 0 |
| S041 | R323 | ST-1818 | IncFII(pSE11), IncHI2, IncHI2A, IncFII, IncFIB(pENTAS01), IncN, ColE10, ColpVC, ColRNAI | *etpD, lpfA* | O116:H16 | 3 |
| S042 | R027 | ST-2705 | IncHI2A, IncHI2, Col(MGD2), p0111, IncX1 | *astA* | O-:H10 | 1 |
| S043 | R040 | ST-4965 | IncI1, IncFII(pHN7A8), IncHI2A, IncHI2, IncFIB(AP001918), IncN, ColRNAI | *astA* | O13:H30 | 2 |
| S044 | R045 | ST-4965 | IncHI2A, IncHI2, IncFIB(AP001918), IncFII(pHN7A8), IncN, IncI2, ColRNAI | *astA* | O13:H30 | 2 |
| S045 | R066 | ST-43 | IncHI2A, IncHI2, IncFIB(AP001918), IncN, IncX1, ColRNAI | *iss* | O6:H10 | 1 |
| S046 | R068 | ST-178 | IncFIB(pLF82), IncHI2, IncHI2A, IncFIA(HI1), IncFII, IncFIB(AP001918), IncX1, p0111, IncI2 | *astA, cma, iroN, iss* | O18:H45 | 1 |
| S047 | R110 | ST-354 | IncFIC(FII), IncHI2, IncFIB(AP001918), IncN, IncHI2A, IncQ1 | *air, eilA, lpfA* | O154:H51 | 1 |
| S048 | R121 | ST-1818 | IncFII(pSE11), IncHI2, IncHI2A, IncFII, IncFIB(pENTAS01), IncN, IncX1, ColE10, ColpVC, ColRNAI | *etpD, lpfA* | O116:H16 | 2 |
| S049 | R127 | ST-155 | IncHI1B(CIT), IncHI2, IncFIB(K), IncHI2A, p0111, Col(MG828), ColRNAI | *iss, lpfA* | O154:H9 | 2 |
| S050 | R232 | ST-1324 | IncHI2A, IncHI2, IncN, TrfA, p0111, IncX2 | *astA, lpfA* | O116:H20 | 1 |

- Not identified; *air*, Enteroaggregative immunoglobulin repeat protein; *astA*, EAST-1 heat-stable toxin; *cba*, Colicin B; *cma*, Colicin M; *eilA*, Salmonella HilA homolog; *etpD*, Type II secretion protein; *iha*, Adherence protein; *ireA*, Siderophore receptor; *iroN*, Enterobactin siderophore receptor protein; *iss*, Increased serum survival; *lpfA*, Long polar fimbriae; *mchF*, ABC transporter protein MchF; *tsh*, Temperature-sensitive hemagglutinin.
